# P-547. Efficacy and Safety of B/F/TAF in Treatment-Naïve People With HIV Aged ≥ 50 Years: 5-Year Follow-Up From Two Phase 3 Studies

**DOI:** 10.1093/ofid/ofae631.746

**Published:** 2025-01-29

**Authors:** Cissy Kityo, Samir K Gupta, Princy N Kumar, Amy Weinberg, Bhumi Gandhi-Patel, Hui Liu, Jason Hindman, Jürgen Rockstroh

**Affiliations:** Joint clinical Research Center, Kampala, Luuka, Uganda; Indiana University School of Medicine, Indianapolis, Indiana; Georgetown University Medical Center, Washington, DC, USA, Washington, District of Columbia; Gilead Sciences, Inc., Foster City, California; Gilead Sciences, Inc., Foster City, California; Gilead Sciences, Inc., Foster City, California; Gilead Sciences, Foster City, California; Department for Internal Medicine I, University Hospital of Bonn, Bonn, Germany, Bonn, Nordrhein-Westfalen, Germany

## Abstract

**Background:**

An increasing proportion of people with HIV (PWH) are aged ≥ 50 years, with a greater burden of age-related comorbidities; however, long-term analyses of this population are limited. We present key treatment outcomes through 5 years of first-line therapy with bictegravir/emtricitabine/tenofovir alafenamide (B/F/TAF) in PWH ≥ 50 vs < 50 years.

**Figure:**
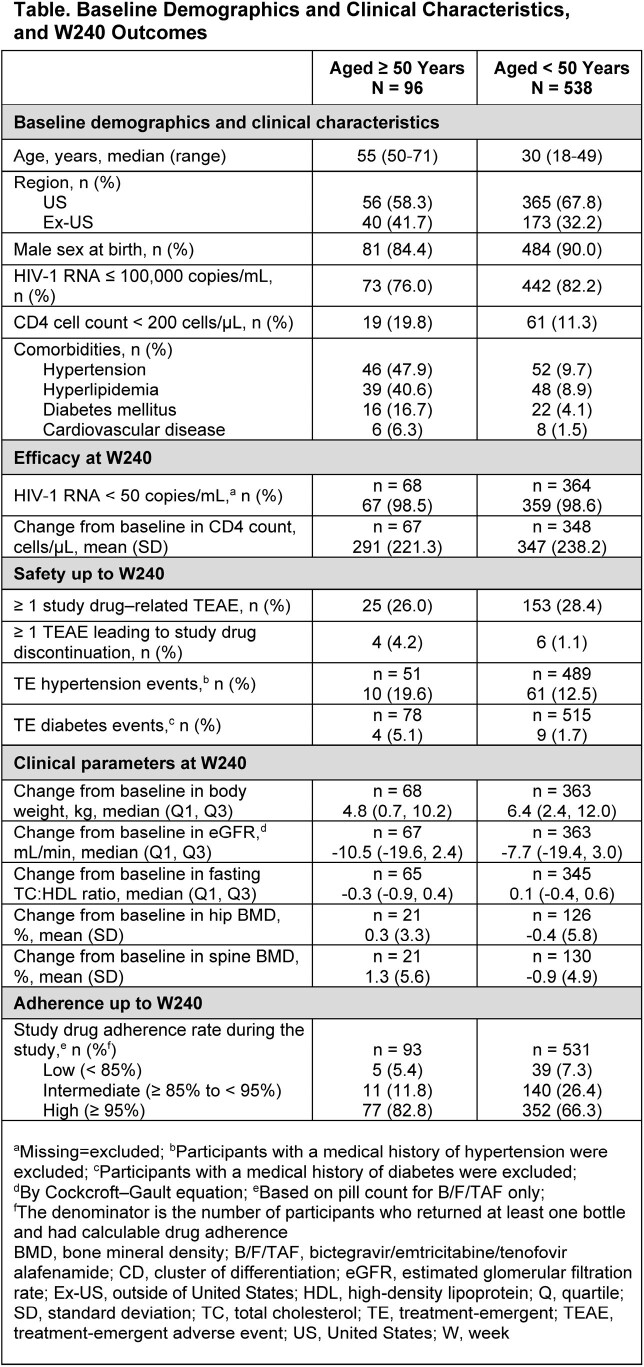

**Methods:**

Studies 1489 (NCT02607930; B/F/TAF vs dolutegravir/abacavir/lamivudine [DTG/ABC/3TC]) and 1490 (NCT02607956; B/F/TAF vs DTG+F/TAF) were randomized, double-blind, multicenter Phase 3 studies in adult PWH. This pooled analysis reports outcomes for participants ≥ 50 vs < 50 years who received B/F/TAF in the 144-week (W) randomization phase and the 96W open-label extension. Baseline demographics and clinical characteristics; proportion of participants with HIV-1 RNA < 50 copies/mL (missing=excluded); adherence; changes in CD4 cell count and metabolic, renal, and bone parameters; and treatment-emergent adverse events (TEAEs) are presented.

**Results:**

Overall, 634 participants received B/F/TAF up to W240; 96 (15.1%) were ≥ 50 years and 538 (84.9%) were < 50 years. Baseline demographics, clinical characteristics, and outcomes are shown in the Table. Higher rates of baseline comorbidities were observed in those aged ≥ 50 vs < 50 years. Both groups had high rates of HIV suppression at W240. A greater proportion of participants aged ≥ 50 vs < 50 years had ≥ 95% adherence (82.8% vs 66.3%; P=0.0015). Changes in CD4 count, weight, eGFR, fasting total cholesterol to high-density lipoprotein ratio, and hip and spine bone mineral density were similar between groups. Proportions of participants with study drug-related TEAEs were similar between groups, with few participants experiencing a TEAE leading to study drug discontinuation. Proportions of TE hypertension and diabetes were modestly higher in the ≥ 50 group vs the < 50 group.

**Conclusion:**

Over 5 years, participants ≥ 50 years were more likely to have high adherence to B/F/TAF treatment vs those < 50 years, with low rates of discontinuations due to AEs in both groups. B/F/TAF maintained high rates of virologic suppression, was well tolerated, and resulted in similar changes in metabolic, renal, and bone parameters in both groups, supporting its use for long-term management of HIV in older PWH.

**Disclosures:**

**Cissy Kityo, MD, MSc, PhD**, Gilead Sciences, Inc.: Medical writing support provided by Aspire Scientific (Bollington, UK)|Janssen Pharma: Grant/Research Support **Samir K. Gupta, MD**, Gilead Sciences, Inc.: Advisor/Consultant|ViiV Healthcare: Advisor/Consultant|ViiV Healthcare: Grant/Research Support **Princy N. Kumar, MD**, Gilead Sciences, Inc.: Advisor/Consultant|Gilead Sciences, Inc.: Board Member|Gilead Sciences, Inc.: Grant/Research Support|Gilead Sciences, Inc.: Medical writing support provided by Aspire Scientific (Bollington, UK)|Gilead Sciences, Inc.: Stocks/Bonds (Public Company)|Johnson & Johnson: Stocks/Bonds (Public Company)|Merck: Advisor/Consultant|Merck: Board Member|Merck: Grant/Research Support|Merck: Stocks/Bonds (Public Company)|Moderna: Stocks/Bonds (Public Company)|Pfizer: Stocks/Bonds (Public Company)|Theratechnologies: Grant/Research Support|ViiV/GSK: Advisor/Consultant|ViiV/GSK: Board Member|ViiV/GSK: Grant/Research Support|ViiV/GSK: Stocks/Bonds (Public Company) **Amy Weinberg, DNP, MS**, Gilead Sciences, Inc.: Employee|Gilead Sciences, Inc.: Stocks/Bonds (Private Company) **Bhumi Gandhi-Patel, PharmD**, Gilead Sciences, Inc.: Employee; Medical writing support provided by Aspire Scientific (Bollington, UK)|Gilead Sciences, Inc.: Stocks/Bonds (Private Company) **Hui Liu, PhD**, Gilead Sciences, Inc.: Employee; Medical writing support provided by Aspire Scientific (Bollington, UK)|Gilead Sciences, Inc.: Stocks/Bonds (Private Company) **Jason Hindman, PharmD, MBA**, Gilead Sciences, Inc.: Employee; Medical writing support provided by Aspire Scientific (Bollington, UK)|Gilead Sciences, Inc.: Stocks/Bonds (Private Company) **Jürgen Rockstroh, Prof**, AbbVie: Advisor/Consultant|AbbVie: Honoraria|Boehringer: Advisor/Consultant|Boehringer: Honoraria|Gilead: Advisor/Consultant|Gilead: Grant/Research Support|Gilead: Honoraria|Gilead Sciences, Inc.: Medical writing support provided by Aspire Scientific (Bollington, UK)|Janssen: Honoraria|Merck: Advisor/Consultant|Merck: Honoraria|ViiV: Advisor/Consultant|ViiV: Honoraria

